# Palliative care needs in Malawi: Care received by people living with HIV

**DOI:** 10.4102/curationis.v39i1.1664

**Published:** 2016-06-29

**Authors:** Esmie Mkwinda, Eucebious Lekalakala-Mokgele

**Affiliations:** 1 Community Department, Kamuzu College of Nursing, University of Malawi, Lilongwe, Malawi; 2 School of Health Care Sciences, Sefako Makgatho Health Sciences University, Pretoria, South Africa

## Abstract

**Background:**

Infection with human immunodeficiency virus (HIV) has changed from an acute to a chronic illness in the past decade, because of highly active antiretroviral therapy (ART). Malawi’s response to the HIV challenge included provision of ART for people living with HIV or AIDS (PLWHA), which significantly reduced HIV- and AIDS-related mortality. In addition, palliative care for PLWHA was introduced as a strategy that improves the success of ART.

**Objective:**

The purpose of the study was to explore the needs of PLWHA concerning care received from primary caregivers and palliative care nurses in Malawi.

**Methods:**

A qualitative, explorative design was used and 18 participants were selected purposefully and interviewed individually using a semi-structured interview guide. Data were analysed using NVivo software package version 10.

**Results:**

Results revealed that PLWHA needed physical care from the primary caregivers due to severity of illness, integration of healthcare services, and continuity of care and proper care from nurses. They also needed knowledge from nurses in several areas which affected decision-making and needed financial and nutritional support.

**Conclusion:**

More could be done in meeting needs of PLWHA to improve their health and survival and assist them to achieve a better quality of life.

## Introduction

The human immunodeficiency virus (HIV) and acquired immune deficiency syndrome (AIDS) remains a threatening pandemic that has eroded and affected many lives requiring palliative care. This pandemic has affected the growth and development of many countries especially in sub-Saharan Africa where the pandemic is increasing and the quality of life of people living with HIV or AIDS (PLWHA) is adversely affected (Uwimana & Struthers 2007:576). Malawi ranks among 10 countries with a high HIV prevalence rate globally (Mwagomba *et al*. 2010:5). Malawi’s response to the HIV challenge included provision of antiretroviral therapy (ART) for PLWHA, which significantly reduced the HIV- and AIDS-related mortality.

The country also provided community home-based care services as recommended by the World Health Organization (WHO) and the Joint United Nations Programme on HIV or AIDS (UNAIDS) to reduce the burden of HIV care on the healthcare system (Bowie, Gondwe & Bowie 2010:370; Nyirenda *et al*. 2006:69; Pindani *et al*. 2013:4). In addition, in 2002 Malawi introduced a palliative care approach for PLWHA, as advocated and recommended by the WHO as a strategy that improves the successful outcome of ART (Jameson 2007:852; WHO 2011).

The goal of palliative care is to assist patients to achieve the best possible quality of life by meeting their perceived and expressed needs (Chui *et al*. 2009:1860). Achieving the palliative care goal requires nurses to care for patients physically, socially, psychologically and spiritually, but also to support the caregivers who provide care to PLWHA at home (Ministry of Health 2008:11). This strategy could be effective if the needs of PLWHA in Malawi are known.

The palliative care approach utilised nurses as the main providers of care at the clinics of selected government hospitals, some private hospitals and non-governmental organisations (NGOs), while home-based care is provided at home by family members who are the primary caregivers (Ministry of Health 2008:13). The home-based care model of palliative care delivery has proved to be cost-effective for long-term care of PLWHA, as it allows patients to be looked after at home by family members since the Malawi Government takes care of all medical expenses (Chinula 2008:3; Pindani 2008:145).

At home family members, who are mostly women, undertake complex tasks in caring for PLWHA, such as symptom assessment and management, hygiene care and medication administration (Dippenaar, Chinula & Selaledi 2011:23; Hudson *et al*. 2008:270). Studies show that the primary caregivers fail to cope with the demands of the illness due to the different needs of PLWHA and multiple, complex care roles they need to undertake (Chinula 2008:45; Hudson *et al*. 2008:270).

Although ART has increased the life expectancy of PLWHA, they still face physical, psychological and spiritual needs which could affect their quality of life, as there is no cure for the condition (Lowther *et al*. 2012:12). According to Nicolau *et al*. (2011:59), palliative care for PLWHA requires a wide range of therapies which includes medical and non-medical care in different settings to ensure a better quality of life for both the patient and their families.

It is significant to know the needs of PLWHA for proper care to be provided, as infection with HIV is now considered a chronic condition which requires care from different settings such as ART clinics, palliative care clinics, hospitals and at home. A study conducted in Malawi showed that palliative care nurses work closely with patients, but have needs for training, resources, support and guidelines, which is affecting the care provided to PLWHA (Mkwinda, Sengane & Lekalakala-Mokgele 2014:441). Since palliative care was introduced in Malawi, the needs of PLWHA treated by palliative care nurses and primary caregivers have not been assessed. Therefore, this study explored the needs of PLWHA concerning care from primary caregivers and palliative care nurses in palliative care in Malawi.

## Research design and method

### Design

A qualitative, contextual, descriptive and explorative study design was used in this study to collect information on needs of PLWHA in relation to care received from primary caregivers and nurses. The nature of this approach allowed the researcher to gain an in-depth understanding of the phenomenon provided by the participants.

### Population and sampling technique

The population comprised of all (1154) the PLWHA who were receiving treatment from the three palliative care clinics in Malawi. A purposive sampling technique was used, with no predetermined number of participants. The inclusion criteria comprised of all the PLWHA on a palliative care programme at the three palliative care clinics, with a minimum of three months of receiving the services. Both genders were included in the study, and respondents had to be at least 18 years of age.

A purposive sample of 18 participants was selected: 7 from Ndimoyo palliative care clinic, 5 from Nkhoma palliative care clinic, and 6 from Kamuzu Central Hospital palliative care clinic.

### Pilot study

The semi-structured in-depth interview guide was pilot tested using two individual participants at Ekwendeni Hospital palliative care clinic in order to increase credibility and adapt the tool accordingly. Data from the pilot study were not included in the main study.

### Data collection

A semi-structured interview guide was used to conduct individual in-depth interviews with participants. The semi-structured interview guide had one main question and some probes to facilitate collection of data, and was constructed in English and translated into Chichewa, the indigenous language of Malawi. The information obtained from the participants was tape recorded, and the interviews took 45–60 min each. The data collection tool explored the needs of PLWHA in relation to the care received from primary caregivers and palliative care nurses in palliative care in Malawi. It also comprised a demographic profile of the participants.

### Data analysis

Data analysis was done concurrently with data collection until data saturation was reached and no new information emerged. Firstly, data were manually analysed using Tesch’s eight steps and then transported into NVivo software package version 10, where themes were identified (Creswell 2009:185–189).

### Setting of the study

The study was conducted at three sites. These were Ndimoyo palliative care centre in Salima district, which is referred to as group one (G1); Kamuzu Central Hospital palliative care clinic, referred to in this study as group two (G2); and Nkhoma palliative care clinic, referred to in this study as group three (G3), all of which were in Lilongwe district. These sites represented NGO, government and private palliative care delivery in Malawi, respectively.

### Trustworthiness

The study applied principles of trustworthiness as outlined by Lincoln and Guba (1994, cited in Polit & Beck 2008). Credibility was achieved by piloting of the data collection tool and member checking, where data findings were confirmed using follow-up questions with participants. Prolonged engagement with the data during data collection and analysis allowed the researchers to have an in-depth understanding and to identify recurring themes. Utilising the services of a skilled independent coder during data analysis ensured that dependability was achieved. Audio-recorded data ensured confirmability, and transferability was achieved by the triangulation of study sites to obtain multiple views of participants. Authenticity was achieved by using participants’ quotes when presenting the findings.

### Ethical considerations

Permission to conduct the study was obtained from Sefako Makgatho University Research and Ethics Committee (formerly known as University of Limpopo MEDUNSA campus) in South Africa (Reference number: MREC/H/ 13/2012:PG), the National Health Sciences Research Committee of Malawi (Reference number: NHSRC992), the District Health Officers from Lilongwe and Salima Districts and management of the three sites. Voluntary participation and informed consent, confidentiality, anonymity, protection from harm, veracity and non-discrimination were maintained throughout the study.

## Results

The results include demographic information of the 18 participants which comprises age, sex, religious background, marital status, level of education and occupation. It also includes the discussion of themes and categories that emerged from the data.

### Demographic characteristics of participants

The demographic characteristics of the 18 PLWHA who were purposively selected from the three study sites are presented in Table 1.

**TABLE 1:** Demographic data of the 18 participants interviewed.

Variable	No.	%
**Age (years)**		
18–25	1	5.5
26–30	3	16.6
31–40	6	33.3
41–50	6	33.3
51–60	2	11.1
**Sex**		
Female	9	50
Male	9	50
**Religious background**		
Christian	7	38.9
Muslim	11	61.1
**Marital status**		
Married	14	77.7
Widowed	2	11.1
Divorced	1	5.5
Single	1	5.5
**Level of education**		
No formal education	3	16.6
Primary education	10	55.5
Secondary education	3	16.6
Tertiary education	2	11.1
**Occupation**		
Farming	5	27.7
Primary school teachers	4	22.2
Small-scale businesses	3	16.6
Unemployed	6	27.7

*Source*: Authors’ own work

Five themes emerged from the individual interviews relating to needs of the PLWHA, namely, physical care from primary caregivers, quality healthcare services, knowledge, resources and support. The categories within the themes are shown in Table 2.

**TABLE 2:** Themes and categories that emerged.

Themes	Categories
Need for physical care from primary caregivers	Need for assistance with bathing Need for assistance with mobility and changing positions in bed Need for assistance with wound care
Need for quality healthcare services	Need to access all healthcare services from one clinic Need for prompt treat-ment Need to be treated with compassion
Need for knowledge	Knowledge of ART Knowledge of cancer of the cervix Knowledge on sexual health in sero-discordant relation-ships Knowledge on breastfeed-ing Healthcare knowledge of primary caregivers
Need for resources	Need for infrastructure Need for financial re-sources Need for good nutrition
Need for support	Support in form of commu-nication Need for peer support Support by reducing stigma in the community Need for spiritual support

*Source*: Authors’ own work

### Theme one: Need for physical care from primary caregivers

The findings highlighted that some participants depended on primary caregivers for assistance with self-care due to the severity of their illness. Under this theme, three categories emerged: assistance with bathing; assistance with mobility and changing positions in bed; and assistance with wound care.

#### Assistance with bathing

Several reasons were revealed during the individual interviews for the need for assistance with bathing, which included being too weak and wanting to feel comfortable and accepted:

‘… I fail to bath myself … I need help … my caregiver assists me with this’ (Participant (P)3, G1);

‘… she would bathe me which made me feel clean and accepted…’ (P2, G2);

‘… she assists me with bathing because I am weak…’ (P5, G3).

#### Assistance with mobility and changing positions in bed

Some participants cited how their primary caregivers assisted them with mobility and changing positions in bed to prevent pressure sores and numbness. Others mentioned the need for exercises such as walking, which primary caregivers assisted them with, as evident in the following quotes:

‘… at least I can walk now which is a very important feature in my life and for my survival … my caregiver has helped me in this … I could have been worse…’ (P2, G1)

‘When I was bedridden, my daughter used to turn me to prevent sores and numbness … this is important.’ (P 1, G2)

‘I was taught to exercise by walking in order to gain back my strength … I cannot walk alone without assistance … my caregiver helps me….’ (P3, G2)

### Assistance with wound care

Two participants who had wounds shared that their primary caregivers and nurses dressed their wounds, which brought comfort and healing:

’I have a wound on the back of my right leg … I fail to dress it myself … my wife does it and this brings comfort, she also tells me how the wound is looking.’ (P6, G3)

’Nurses dress my wound every second day at the clinic … this is helping it to heal.’ (P4, G1)

### Theme two: Need for quality healthcare services

Three categories emerged from this theme, which pertains to the need to access all healthcare services from one clinic; prompt treatment; and being treated with compassion.

#### Need to access healthcare services from one clinic

Even though participants accessed healthcare services from several clinics, they shared that they would benefit greatly from a one-stop clinic where all services are integrated. This would ensure that continuity and monitoring of care is achieved, with less time and money spent visiting different clinics:

‘I get ART from the clinic next door and palliative care services from this clinic, I use different files and different days, sometimes you are given the same medications twice … they could provide the services at one clinic to treat all my needs at once….’ (P4, G3)

‘Some of the medications are duplicated since I use different files at these clinics, they don’t have to work in isolation…. It is hard for nurses to monitor our progress at these different clinics … they need to work together.’ (P2, G1)

#### Need for prompt treatment

Participants indicated that they need prompt treatment from nurses when they visit the clinics, as they spend a lot of time waiting before being attended to:

‘When I go to the government hospital firstly I spend the whole day there, the nurses come at their own time. I was there two days ago, I arrived at 8am and was only helped around 2pm and they do not even care.’ (P5, G1)

‘There is normally only one nurse helping patients and they only use this room … you spend hours before your turn comes. I wish that there were more nurses assisting us especially if you are in pain, you are not helped quickly….’ (P6, G3)

‘… they try their best to assist us, the nurses are so few and they take time to see each patient … there’s need for more nurses so that we get assisted quickly….’ (P2, G1)

#### Need to be treated with compassion

Some participants were concerned about the way some nurses assisted them at some clinics. They commented that although some nurses were considerate and caring, others failed to treat them with respect and compassion:

‘… the way nurses treat you and talk to you says it all … they also seem to be too busy when they are really not, … one nurse once said to me, was I there when you contracted your disease? So please don’t trouble me with your complaints … this needs to change especially from people who have the knowledge of our condition….’ (P4, G1)

‘… you get two kinds of nurses, some are nice and they really care and want to know what you are going through and help while others treat us to finish the queue … they shout anyhow, have a bad attitude towards us, and I wish that they could know how we feel.’ (P2, G2)

### Theme three: Need for knowledge

Five categories emerged from this theme, namely: ART knowledge; cancer of the cervix; prevention of infection in sero-discordant relationship; breastfeeding; and healthcare knowledge of primary caregivers.

#### Knowledge of ART

Some participants expressed lack of adequate knowledge about ART, which contributed to discontinuation of treatment and noncompliant behaviour:

‘I also need more information on ART … I stopped taking them for six months … I felt better.’ (P6, G2)

‘… I took ART for three months and was fine, but they say we should take them throughout … I don’t see the need … it does not make sense….’ (P4, G3)

#### Knowledge of cancer of the cervix

Some participants in the study had cervical cancer while taking ART and wished to understand the relationship between HIV infection and cancer. They expressed that while on ART they had not expected to be infected by opportunistic infections such as cancer:

‘I used to think that while I am taking ART, I cannot get sick with cancer or things like that … I was told that I have cancer of the cervix and it is in advanced stage … what was I supposed to do before….’ (P3, G3)

‘Nurses should teach patients on conditions such as cervical cancer and screen us, people are suffering with this condition … they only teach you when you are already sick….’ (P1, G1)

#### Knowledge on sexual health in sero-discordant relationship

Participants in a sero-discordant relationship mentioned that they lacked knowledge on prevention of HIV infection, while those wanting to get married also wished to have HIV prevention knowledge:

‘… my wife is HIV negative I don’t know how … nurses should teach us and other families where one of us is HIV negative because I don’t want to infect her.’ (P5, G1)

‘I need knowledge on sexual issues because I want to get married and have children … but I don’t want to infect the child and my partner … they don’t teach us such things at the clinic, it is hard to ask such issues….’ (P3, G2)

#### Knowledge on breastfeeding

Some participants lacked proper information on breastfeeding, which caused confusion and worry to mothers since they wanted to prevent infecting their babies:

‘… some nurses say that we should not breastfeed at all, like what I did with my last child, yet now others are saying that we should breastfeed for six months….’ (P2, G3)

‘… nurses should tell us the right information because now our babies will be HIV positive because they say we should breastfeed them for two years ….’ (P4, G3)

#### Healthcare knowledge of primary caregivers

Participants shared that their primary caregivers needed to be trained in patient care, as lack of this knowledge negatively affected the care that they were receiving:

‘I also wish that my wife should be taught thoroughly about this condition and the caring that she is doing to have reasons than just doing it.’ (P2, G1)

‘… caregivers should also be trained … to give us the right care … real care is not provided.’ (P4, G1)

### Theme four: Need for resources

Three categories emerged from this theme, namely infrastructure, financial and nutrition resources.

#### Need for infrastructure

One participant mentioned the need for a palliative care ward where patients could rest:

’I think our hospital should have a palliative care ward … sometimes I get very sick and here they give you medications and you have to go home without even resting a bit.’ (P1, G2)

#### Need for financial assistance

Participants mentioned the need for money in order for them to afford different needs, such as paying for medications, transportation to the hospital and other significant costs such as school fees for their children:

’The other need is money for transport to the hospital and for buying medications. We are paying for everything recently except for ART. Now everything circles around money … my children are going to school and I have to pay for school fees.’ (P6, G3)

‘At the moment I spend most of my time visiting these clinics, I spend a lot of money on transport because I go on different days. It is only at palliative care clinic where they give us money for transport.’ (P1, G1)

#### Need for good nutrition

Two participants highlighted the need for proper nutrition with all food groups; others shared the need for nutritional supplements:

‘I wish for proper nutrition with all food groups … we don’t have this at home….’ (P1, G1)

‘I wish for the clinic to assist me with nutritional supplements like soya flour.’ (P6, G2)

‘… they told me about the food that I must eat but … I don’t have money to buy it….’ (P4, G3).

### Theme five: Need for support

Five categories emerged from this theme, namely: need for support in the form of communication; peer support; and support from the church, the community and nurses.

#### Support in the form of communication

Participants stated that they would benefit from regular communication with palliative care nurses via the telephone. They were concerned that they do not have nurses’ contact numbers to call for guidance and advice and for support of the caregivers:

‘… I get sick often at night and sometimes I wish to have the phone number of someone that I could contact at the clinic for advice and management for such….’ (P5, G1)

‘The other problem is that we are not given any contacts … I wish that a way of communication should be devised, even to call for advice.’ (P6, G3)

#### Need for peer support

Participants reported the importance of having a forum with peers who have the same condition, in order to share experiences and needs:

‘The nurses could also help us to form a support group … at the moment I feel alone with this condition, maybe there are other people in the same situation in my community.’ (P3, G3)

‘I also joined the support group which helps me. At the hospital they do teach me what they know but they do not know what happens in my life at home … all of us in that group are HIV positive and we really encourage each other. We treat each other like family and you feel that you are not suffering alone….’ (P2, G1)

#### Support by reducing stigma in the community

Some participants mentioned the need for support from community members as a means of reducing the stigma they experienced in their own community:

‘… if people know that you are HIV positive they talk about you and stigmatise you. That is why I did not want to disclose my status. … I fear rejection….’ (P5, G3)

‘The village members do not involve me in any village activities, even my wife’s friends stopped visiting her … I wish that things could change.’ (P1, G3)

#### Need for spiritual support

Some participants mentioned that they were encouraged by visits from church leaders and members, while others noted that this would improve their sense of well-being:

‘Sometimes they call our church leader to visit me and pray for me which encourages me.’ (P4, G2);

‘The other support that I need at times is prayers from church and other people….’ (P3, G2).

### Discussion

This study explored the needs of PLWHA in relation to care received from the primary caregivers and palliative care nurses in Malawi. The results of the analysed data show that the majority of participants depended on primary caregivers for physical care during periods of illness, and lack of this care affected their quality of life. Performing day-to-day activities such as maintaining hygiene, eating, dressing and looking after one’s health have been documented by several authors as important determinants of health relating to quality of life; this present a challenge to PLWHA due to the severity of the illness, that require caregivers to provide assistance (Gaidhane *et al*. 2008:1098; Majumdar & Mazaleni 2010:13).

Although care was provided to the PLWHA from different clinics, the findings of this study revealed that participants could benefit from integration of healthcare services such as ART provision, palliative care and reproductive health services. Integration and linking of services for PLWHA could be beneficial for promptness and quality of healthcare delivery with sustained improvement to patients (Orner *et al*. 2008:1220). Kohli *et al*. (2012:1) are of the opinion that care interventions for PLWHA should not operate in isolation, but be embedded into the spheres of health facility, community, workplace and the environment, and also linked up with the family in order to mitigate the impact of the disease.

Participants shared that although some of them were treated well at the healthcare facilities, others experienced bad attitudes and disrespect from some nurses, which affected the care and health of participants. A study conducted by Hassan and Wahsheh (2011:774) found that nurses lacked HIV knowledge, which led to a negative attitude towards HIV patients. In addition, a study conducted by Lam, Chan and Thayala (2011:9) showed more prejudice from nurses and changed practice behaviours after knowing the patient’s HIV status.

Despite the benefits of adequate knowledge of their condition, the researchers found that most participants lacked knowledge and information in some areas which affected their health behaviours and decision-making. Zukoski, Thorburn and Stroud (2011:1507) found that provision of information is a critical component of health literacy and survival of PLWHA which should not be ignored.

Most participants had proper knowledge of ART, although some of these participants lacked thereof, which resulted in discontinuation of treatment. According to Scajiu, Raveis and Selwyn (2009:1534) ART adherence was reported to be interfered with by chaotic life circumstances and side-effects, which call for healthcare workers to teach the caregivers about patients’ medications so that social support is provided and can influence adherent behaviour. It is important to provide proper ART information, since lack of adherence could lead to retroviral resistance, treatment failure and development of drug-resistant strains of HIV (Eldred & Malitz 2007:S1; Fehringer *et al*. 2006:638). Therefore the participants recommended that there is a need for proper knowledge.

Understanding prevention strategies for partners in discordant relationships and sexual health knowledge were indicated to be lacking in participants. Fakoya *et al*. (2008:683) suggested that HIV-positive men and women and their partners, as well as sero-discordant couples, should be counselled on risk-reduction strategies for natural conception in order to know the risks of HIV transmission so that they make an informed choice. In addition, reproductive clinical care for PLWHA should address contraceptive use, planned pregnancy and protected sex to improve patient empowerment in decision-making (Malta *et al*. 2010:480).

Participants also wished to understand the relationship between cancer of the cervix and ART since they were diagnosed with the condition in advanced stages without being screened. According to Pollack, Balkin and Denny (2006:334), HIV-positive women have a higher risk of human papillomavirus-related disease such as cervical cancer due to their compromised immune system. In addition, limited and controversial data exist on the effect of highly active antiretroviral therapy (HAART) on the natural history of disease such as cancer and recommend that management of women should be the same whether they are on ART or not (Fakoya *et al*. 2008:683). Jedy-Agba and Adebamowo (2012:7) and Myer, Morroni and Rebe (2007:282) suggest that as cancers tend to present at advanced stages with limited treatment options in resource-constrained settings, preventive and early-detection measures such as health education and annual screening should be included in the management of PLWHA as routine.

Participants also shared that their primary caregivers lacked knowledge of their caring role. This finding was consistent with what Majumdar and Mazaleni (2010:13) found – that most primary caregivers who provide care to PLWHA at home lacked knowledge which affected care to patients. Hudson *et al*. (2012:10) argue that caregiver capacity needs enhancement by healthcare professionals in order for them to provide quality care and maintain their health.

The researchers’ findings reveal that most participants reported a lack of resources in the form of infrastructure, financial and nutritional resources, which affected their health and the way they were cared for. According to Agbonyitor (2009:303) and Zerfu *et al*. (2012:11), everything around HIV infection is tied to money, because of the need to pay for children’s school fees, hospital bills and transport, and to buy food. In addition to financial resources, the findings of the study revealed the need for proper nutrition for PLWHA. Various authors have noted that poor nutrition in HIV patients was due to food shortages, and this affects nutritional status and progression of HIV infection in PLWHA (Ahoua *et al*. 2011:14; Fan *et al*. 2011:287; Oguntibeju, Van den Heever & Van Schalkwyk 2007:4327). Kalichman *et al*. (2010:633), Duran *et al*. (2008:346) and Ahoua *et al*. (2011:14) noted that increasing access to food and improving nutrition for PLWHA could have a significant effect on their health and should therefore be integrated into their care and treatment package.

Study participants also revealed the need for support in the form of communication, support from peers, the church, community and nurses for their health to be promoted. Participants wished to have regular communication with nurses. According to McIlfatrick (2007:84), the need for improved coordination and communication on a 24-h basis is beneficial to the patients for continuity of care and reduction of inappropriate hospital admissions. In a systematic review conducted by Van Velthoven *et al*. (2012:5), it was observed that telephonic interventions offered the potential to improve the health of PLWHA cost-effectively and also improve access to healthcare services, even in resource-limited healthcare settings.

Peer support was available for some who benefitted from it, and lacking for others, and was viewed as an important support strategy by participants. A study conducted by Cassidy (2010:1600) in Gambia found that HIV support groups provide a sense of having problems in common which eases the mind, as well as providing company and a circular relationship between the support that is offered and which members themselves provide for each other. Support from the church was also viewed as an important element for participants in this study. In agreement, Watt *et al*. (2009:389) state that religious engagement plays an important role in the lives and health of PLWHA.

## Limitations of the study

The results of this study should be interpreted with caution as only three palliative care facilities were sampled in Malawi; as such, the results should be transferred with caution. Future studies should explore the needs of the primary caregivers and include more healthcare facilities.

## Conclusion

The findings of this study highlight the needs of PLWHA in relation to care received from primary caregivers and palliative care nurses. The physical care needs of participants were provided by primary caregivers, although the study showed that most of them lacked knowledge. The lack of knowledge of their treatment, cancer of the cervix and preventing infecting partners in a relationship and their babies underscores the importance of improving their health and quality of life through communication and resources which include information.

The need for quality healthcare services and resources was mentioned by participants, and these are also vital for the survival and quality of life of PLWHA. It is crucial to meet the needs of PLWHA through education and information, resource provision and support in order for proper care to be provided.

### Recommendations

These recommendations are based on findings of this study, and are as follows:

Primary caregivers should be trained and provided with information on the care of their patients. They should also be provided with refresher workshops to ensure that they have adequate knowledge on caring for PLWHA.

The primary caregivers should be provided with nurses’ contact details and be visited at home on regular intervals as a support measure.

Nurses should conduct a needs assessment on each patient for proper care and support.

All palliative care nurses should be provided with training sessions designed to prepare them for care provision to the patients and support of primary caregivers in all areas, using the following strategies: mentorship by an experienced registered nurse, workshop sessions at specific intervals and peer support from fellow nurses.

Patients should be educated on their condition, the treatment that they are taking and the relationship of their condition to others such as cancer of the cervix; they must also be educated on breastfeeding options to prevent infecting their baby.

Patients in sero-discordant relationships should be taught about how to prevent infection of their partners.

Healthcare facilities should provide resources for care provision of the patient, which should consider infrastructure, financial and nutritional resources.

The Ministry of Health should integrate healthcare services for PLWHA, so that they receive services at a one-stop clinic to achieve continuity of care.

Nurse managers should ensure that adequate staffing is available at healthcare facilities for prompt and proper care provision.
